# Doege-Potter Syndrome: A Solitary Fibrous Tumor Causing Non-Islet Cell Tumor Hypoglycemia

**DOI:** 10.1210/jcemcr/luae017

**Published:** 2024-02-23

**Authors:** Khalid Sheikh, Avni Mody, Alex B Haynes, Pratima Kumar

**Affiliations:** Department of Internal Medicine, Dell Medical School at the University of Texas at Austin, Austin, TX 78712, USA; Department of Internal Medicine, Division of Endocrinology, Dell Medical School at the University of Texas at Austin, Austin, TX 78712, USA; Department of Surgery and Perioperative Care and Department of Oncology, Dell Medical School at the University of Texas at Austin, Austin, TX 78712, USA; Department of Internal Medicine, Division of Endocrinology, Dell Medical School at the University of Texas at Austin, Austin, TX 78712, USA

**Keywords:** Doege-Potter syndrome, non-islet cell tumor hypoglycemia, NICTH, high-molecular-weight IGF-2, big IGF-2, solitary fibrous tumor

## Abstract

Doege-Potter syndrome occurs when incompletely processed insulin-like growth factor 2 (IGF-2), also known as *big IGF-2*, is produced by a solitary fibrous tumor (SFT) and results in non-islet cell tumor hypoglycemia (NICTH). We discuss here the case of a 66-year-old male who presented with a 2-week history of increasing confusion and a serum glucose of 34 mg/dL. The patient's symptoms immediately improved with dextrose. The patient did not use insulin, serum sulfonylurea screen was negative, and testing for adrenal insufficiency was unremarkable. Outpatient laboratory evaluation revealed a serum glucose of 48 mg/dL along with low insulin, C-peptide, and proinsulin levels. Further work-up showed an IGF-2 to IGF-1 ratio of 38:1. A ratio greater than 10:1 is diagnostic of NICTH. Imaging demonstrated a 21-cm mass in the lower abdomen and pelvis. The patient underwent surgical resection. The hypoglycemia resolved immediately postoperatively. Surgical pathology revealed a malignant SFT. In NICTH, big IGF-2 forms a complex that is biologically active and saturates the insulin and IGF receptors, resulting in refractory hypoglycemia. Although glucocorticoids can mitigate hypoglycemia, complete surgical resection is the only definitive treatment of NICTH. This case highlights the importance of maintaining a broad differential for seemingly simple hypoglycemia.

## Introduction

Evaluation of hypoglycemia is warranted in the presence of Whipple triad: symptoms of hypoglycemia (such as lightheadedness, diaphoresis, and tachycardia), a blood glucose of < 55 mg/dL (< 3.0 mmol/L) during the symptoms, and the resolution of symptoms with administration of glucose (1). Hypoglycemia can be due to exogenous or endogenous production or secretion of insulin. In addition, there are non-insulin-mediated causes of hypoglycemia, such as non-islet cell tumor hypoglycemia (NICTH). Although NICTH is rare, it is important to consider this diagnosis when the evaluation of hypoglycemia does not indicate an increased presence of elevated insulin levels and necessitates further investigation to identify and treat the underlying cause.

## Case Presentation

A 66-year-old male presented to the emergency department with confusion and lethargy for 2 weeks. Per family report, the patient was in his usual state of health until 1 year ago when he started having episodes of near syncope with disorientation and slurred speech. He noted that his symptoms improved on ingestion of high carbohydrate meals and needed to consume food every 4 hours to prevent these symptoms. He presented to the emergency department because of the increased frequency of these episodes.

On admission, magnetic resonance imaging (MRI) stroke protocol was performed and did not reveal any abnormalities. Serum glucose was noted to be 34 mg/dL (1.9 mmol/L) (reference range, 70-100 mg/dL, 3.9-5.6 mmol/L). Upon administration of dextrose, the patient's symptoms immediately improved. No other significant electrolyte abnormalities were noted. The patient denied taking any over-the-counter medications, insulin, or sulfonylureas. The patient did not have a history of diabetes, kidney disease, or liver disease.

Further evaluation during the admission revealed a negative urine sulfonylurea screen. Results of a standard dose (250 mcg) ACTH stimulation test were unremarkable, with cortisol 20.9 mcg/dL (576.6 nmol/L) at 30 minutes and 26.1 mcg/dL (720 nmol/L) at 60 minutes. In addition, laboratory evaluations did not reveal elevated levels of insulin, C-peptide, or proinsulin when the serum glucose was 48 mg/dL (2.7 mmol/L). The patient was discharged on hydrocortisone 20 mg in morning and 10 mg in the afternoon by the hospitalist team. During the hospital follow-up visit with his primary care physician, the patient was found to have a serum blood glucose of 31 mg/dL (1.7 mmol/L) and was referred to Endocrinology.

## Diagnostic Assessment

**Figure 1.**
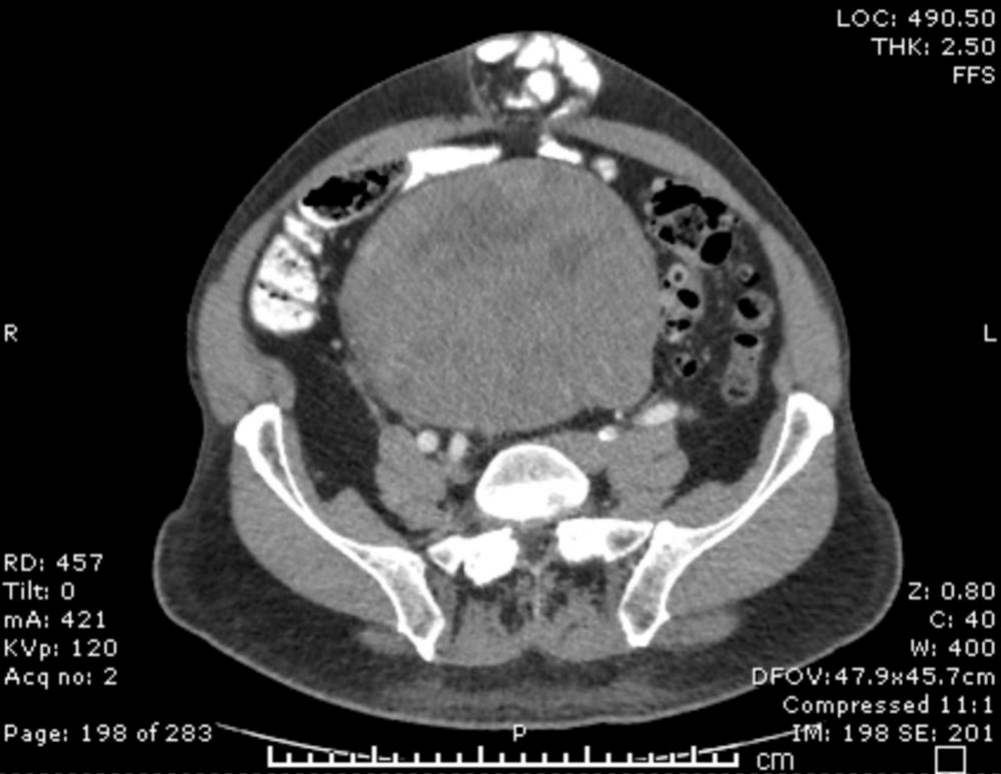
Computed tomography of abdomen and pelvis. Large mass in the lower abdomen and pelvis measuring 20.5 cm.

Upon establishing care with our endocrinologist, a continuous glucose monitor (CGM) was placed, which demonstrated that > 10% of time was spent below 70 mg/dL (3.9 mmol/L) in a 14-day period. Outpatient laboratory evaluation revealed a negative urine sulfonylurea screen and did not reveal elevated levels of insulin, C-peptide, or proinsulin in the setting of spontaneous hypoglycemia, thus ruling out insulinoma. At this time, the suspicion for non-insulin-mediated processes for hypoglycemia was high on the differential. Serum IGF-2 and IGF-1 levels, which were ordered at Quest Diagnostics, demonstrated a significantly elevated IGF-2 level and ratio of IGF-2 to IGF-1, which is diagnostic of non-islet cell tumor hypoglycemia (see Table 1). Subsequently, a computed tomography (CT) of the abdomen and pelvis was performed, which demonstrated a 20.5-cm heterogeneous, complex mass in the lower abdomen and pelvis (see Fig. 1). This mass was located superior to and compressed the urinary bladder. The patient reported no abdominal complaints despite this large mass. Biopsy of the mass was performed and demonstrated a fibrohistiocytic mesenchymal proliferation with expression of STAT-6 and beta-catenin. These findings were diagnostic of a solitary fibrous tumor (SFT). Glucocorticoid therapy was continued and pre-meal acarbose was added. The patient was diagnosed with NICTH and was referred to surgical oncology.

**Table 1.** Laboratory blood values during spontaneous hypoglycemia

Compound (normal range)	Outpatient follow-up
Glucose (70-100 mg/dL, 3.9-5.6 mmol/L)	48 mg/dL, 2.7 mmol/L
C-peptide (1.1-4.4 ng/mL, 0.4-1.5 nmol/L)	0.3 ng/mL, 0.1 nmol/L
Insulin (2.0-24.9 uU/mL, 20.8-173.6 pmol/L)	< 0.4 uU/mL, < 2.8 pmol/L
Proinsulin (1.4 uU/mL, 0-10 pmol/L)	0.5 uU/mL, 3.5 pmol/L
Sulfonylurea	Negative
IGF-1 (41-279 ng/mL, 13.6-92.4 nmol/L)	31 ng/mL, 10.3 nmol/L
IGF-2 (333-967 ng/mL, 110.3-320.2 nmol/L)	1199 ng/mL, 397.0 nmol/L

## Treatment

**Figure 2.**
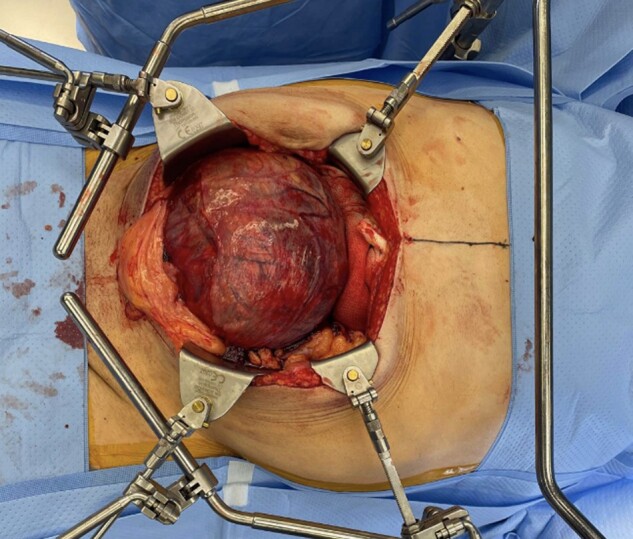
Tumor resection. Gross specimen shows a well encapsulated mass measuring 23.5 cm.

**Figure 3.**
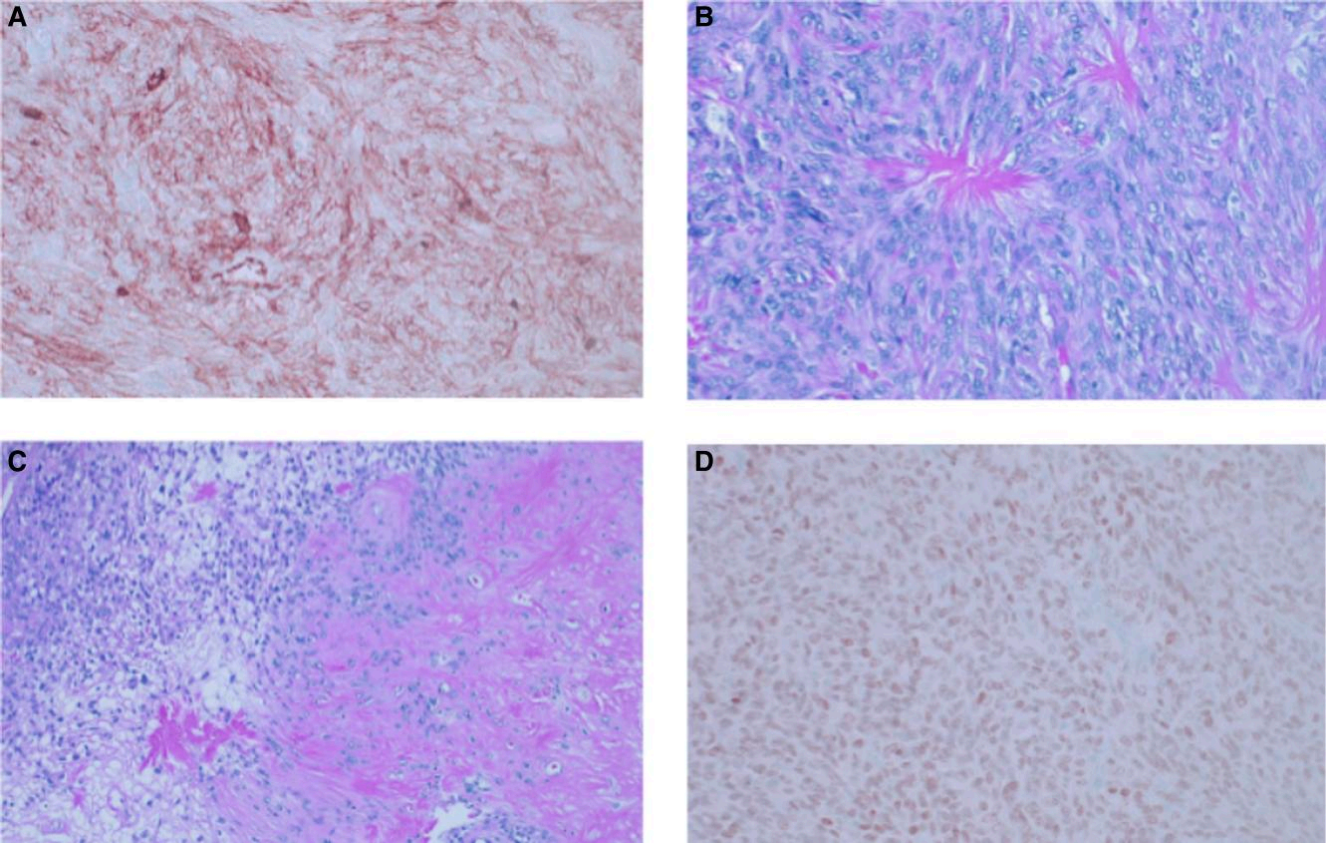
Panel A, CD-34 staining was diffusely positive. Panel B, C, Hematoxylin and eosin staining with evidence of necrosis. Panel D, STAT-6 stain was diffusely positive.

The patient underwent surgical resection of the pelvic tumor with en bloc resection of the vas deferens and the posterior bladder wall and bilateral ureteral stent placement by the surgical oncology and the urology team. The mass measured 23.5 cm and weighed greater than 2.2 kg (see Fig. 2). Surgical pathology demonstrated a histologic grade 3 solitary fibrous tumor with 10% necrosis staining positive for CD-34, beta-catenin, and STAT-6 (see Fig. 3).

## Outcome and Follow-Up

The patient's blood glucose levels normalized in the immediate postoperative period. He was discharged on a physiologic dose of hydrocortisone with a plan to taper as an outpatient, since he had been on glucocorticoids for 1 year. During his 6-week follow-up, the patient denied having any episodes of hypoglycemia and was grateful that he can sleep during the night without having to wake up for food. Postoperative IGF-2 level decreased to 622 ng/mL (205.9 nmol/L).

## Discussion

Non-islet cell tumor hypoglycemia (NICTH) is a rare paraneoplastic syndrome in which non-insulin-mediated hypoglycemia occurs as a result of tumoral overproduction of an incompletely processed insulin-like growth factor 2 (IGF-2), also known as *big IGF-2* or *high-molecular-weight IGF-2*. Doege-Potter syndrome is characterized by the production of big IGF-2 specifically from SFTs, which are rare soft tissue masses of mesenchymal origin (2).

SFTs account for only 3.7% of soft tissue sarcomas. These tumors arise from connective tissue and are most often found in the pleura, retroperitoneum, or abdominal cavity. Metastasis occurs in 10% to 15% of SFTs (3). Immunohistochemistry demonstrating STAT-6 expression can confirm the diagnosis of an SFT (4). A key feature of SFTs is their associated *NAB2-STAT6* fusion gene that drives the dysregulation of IGF-2, leading to abnormal processing of IGF-2 precursors and excess production of big IGF-2.

Typically, IGF-2 is mainly bound to IGF-binding protein 3 (IGF-BP3) and the acid-labile subunit, forming a large ternary complex. As this complex has a high molecular mass, it is unable to cross the endothelial barrier. A smaller percentage of IGF-2 is bound only to IGF-BP3, forming a binary complex. This binary complex can cross the endothelial barrier and exert its effects. In NICTH, big IGF-2 is only able to bind to IGF-BP3, forming the binary complex that is biologically active and saturates the insulin and IGF receptors (5). This leads to an increase in glucose consumption by the tissues, decrease in glucagon secretion, decrease in glycogenolysis, and decrease in gluconeogenesis, resulting in refractory hypoglycemia (6). In addition, the increase in IGF-2 activity suppresses insulin, growth hormone, and consequently IGF-1. As the assay for big IGF-2 is not commercially available, an IGF-2 to IGF-1 ratio greater than 3 is suggestive of the diagnosis of NICTH, and a ratio greater than 10 is diagnostic (7).

Complete surgical resection is the only definitive treatment of these SFTs, thus highlighting the importance of detecting these tumors as early in their course as possible before metastasis can occur. If surgery is delayed or not feasible, ongoing management includes increasing caloric intake and tumor debulking if it is not resectable.

Glucocorticoid therapy has been demonstrated in many cases to be the most effective form of symptomatic therapy. Steroid doses equivalent to more than prednisone 20 mg daily have been demonstrated to be effective in treating hypoglycemia, in part by suppressing IGF-2 production. Recombinant human growth hormone (rhGH) has also been shown to be effective in treating hypoglycemia by stimulating hepatic gluconeogenesis and glycogenolysis. However, as rhGH has the potential to increase tumor growth, its use may not be preferred (8). The potential mortality from not only the malignancy but also from the progressing hypoglycemia with tumor growth demonstrates the importance of having a high clinical suspicion for a paraneoplastic syndrome in a patient presenting with non-insulin-mediated hypoglycemia.

## Learning Points

- Hypoglycemia can be classified as insulin-mediated or non-insulin-mediated.
- NICTH should be suspected in patients with a non-insulin-mediated hypoglycemia.
- Doege-Potter syndrome occurs when there is an overproduction of incompletely processed IGF-2 specifically from solitary fibrous tumors.
- Big IGF-2 can activate the insulin and IGF receptors, leading to profound hypoglycemia.
- Glucocorticoids are often used to treat the symptoms of hypoglycemia, but complete surgical resection is the only curative treatment.

## Contributors

All authors made individual contributions to authorship. P.K. and A.M. were involved in the diagnosis and work-up of the patient and contributed to the writing of the discussion. A.H. was involved in the surgical component of the case and assisted in contributing to the surgical aspects of the case presentation. K.S. organized the writing of the entire case presentation.

## Data Availability

Original data generated and analyzed during this study are included in this published article.

## Abbreviations

IGF: insulin-like growth factor
IGF-BP3: insulin-like growth factor binding protein 3
NICTH: non-islet cell tumor hypoglycemia
SFT: solitary fibrous tumor
